# Structural Dynamics of DPP-4 and Its Influence on the Projection of Bioactive Ligands

**DOI:** 10.3390/molecules23020490

**Published:** 2018-02-23

**Authors:** Simone Queiroz Pantaleão, Eric Allison Philot, Pedro Túlio de Resende-Lara, Angélica Nakagawa Lima, David Perahia, Maria Atanassova Miteva, Ana Ligia Scott, Kathia Maria Honorio

**Affiliations:** 1 Center for Natural and Human Sciences, Federal University of ABC, 09210-170 Santo André, SP, Brazil; simone.queiroz@ufabc.edu.br (S.Q.P); pedro.lara@ufabc.edu.br (P.T.d.R.-L.); angelica.lima@ufabc.edu.br (A.N.L.); 2 Center for Mathematics, Computing, and Cognition, Federal University of ABC, 09210-170 Santo André, SP, Brazil; ericphilot@gmail.com (E.A.P.); analigiascott@gmail.com (A.L.S.); 3 École Normale Supérieure Paris-Saclay, Laboratory of Biology and Applied Pharmacology, 94235 Cachan, France; david.perahia@ens-cachan.fr; 4 Inserm UMR-S 973-Paris Diderot University, Therapeutic Molecules by in silico approaches, 75013 Paris, France; maria.miteva@paris7.jussieu.fr; 5 Department of Computational & Systems Biology School of Medicine, University of Pittsburgh, 15260 Pittsburgh, PA, USA; 6 School of Arts, Sciences and Humanities, University of Sao Paulo, 03828-0000 Sao Paulo, SP, Brazil

**Keywords:** diabetes, DPP-4, functional movements, normal modes, molecular dynamics, binding sites, molecular interactions

## Abstract

Dipeptidyl peptidase-4 (DPP-4) is a target to treat type II diabetes mellitus. Therefore, it is important to understand the structural aspects of this enzyme and its interaction with drug candidates. This study involved molecular dynamics simulations, normal mode analysis, binding site detection and analysis of molecular interactions to understand the protein dynamics. We identified some DPP-4 functional motions contributing to the exposure of the binding sites and twist movements revealing how the two enzyme chains are interconnected in their bioactive form, which are defined as chains A (residues 40–767) and B (residues 40–767). By understanding the enzyme structure, its motions and the regions of its binding sites, it will be possible to contribute to the design of new DPP-4 inhibitors as drug candidates to treat diabetes.

## 1. Introduction

Type II diabetes mellitus, a chronic metabolic disease related to hyperglycemia, has a global estimate of reaching 642 million cases by the year 2040 [1]. The inhibition strategy of the dipeptidyl peptidase-4 (DPP-4) enzyme is currently employed in the treatment of this disease. However, some substances available on the market can cause anemia, neuropathic risk, pancreatitis and nausea [2]. To understand these side effects, an accurate analysis of the structural characteristics of this biological target is required, since its mechanism of action is not yet fully elucidated. Therefore, understanding the functional motions of the enzyme may contribute to the projections of new and effective drug candidates with fewer side effects.

**Figure 1:**
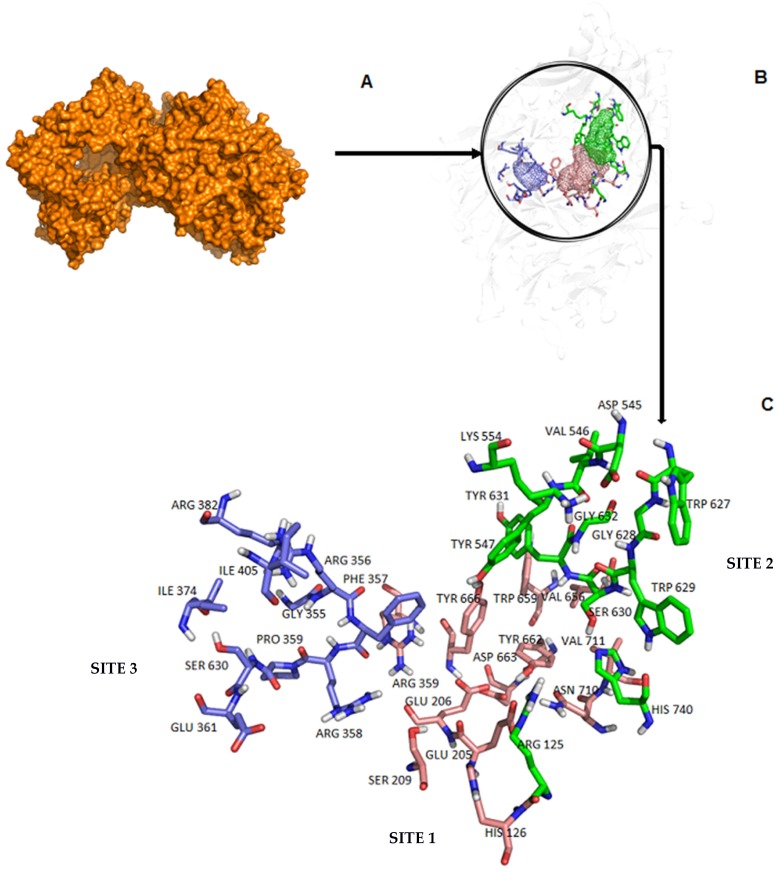
(**A**) Dimeric representation of the DPP-4 enzyme (PDB code: 4A5S [14,15]); (**B**) Representation of three binding sites of the DPP-4 enzyme found by FTSite [18,19,20] and FTMap [18,20,21,22] where regions 1 and 2 (colored in salmon and green, respectively) correspond to the active sites described in the literature (sites 1 and 2) and region 3 (colored in blue) is an alternative binding site (site 3) not described in the literature. (**C**) Key residues of the active site of DPP-4. Region 3 is a possible candidate as an allosteric binding site.

The enzyme DPP-4 is classified as a glycoprotein, composed of three distinct regions: (i) cytoplasmic-residues from 1 to 6; (ii) transmembrane-residues 7 to 29 and (iii) extracellular-residues 30 to 766. Its most important catalytic function is the degradation of incretins such as glucagon-like peptide-1 (GLP-1) and gastric inhibitory polypeptide (GIP), in which metabolic regulation contributes to the control of glucose levels in the blood [3]. Non-catalytic functions of DPP-4, such as T cell co-stimulation, depend on the interaction with another protein known as adenosine deaminase (ADA) [4]. The active site of DPP-4 is divided into sub-regions: a catalytic triad consisting of Ser630, Asp708 and His740; an oxyanion cavity containing Tyr47 and Ser631; a region with saline bridging residues such as Glu205, Glu206 and Tyr662. The pockets S1 and S2 contain Arg125, Ser209, Phe357, Arg358, Tyr547, Ser631, Val656, Trp659, Tyr62, Tyr666, Asn710, and Val711 as illustrated in Figure 1 (region of interest for the action of DPP-4 inhibitors) [5,6,7,8,9,10,11]. Another (non-catalytic) binding region that interacts with adenosine deaminase complexing protein 2 (ADA) consists of the following residues: Asn281, Leu294, Leu340, Val341, Ala342, and Arg343 [12,13], but it is not of interest in devising new inhibitors.

In this study, we applied normal mode analysis to study the functional motions of DPP-4 in four cases: (i) a system containing an inhibitor only in the A chain (residues 40–767) of the protein; (ii) containing an inhibitor only in the B chain (residues 40–767) of the protein; (iii) with an inhibitor in both chains, and (iv) without an inhibitor in both chains. We used 6-[(3*S*)-3-aminopiperidin-1-yl]-5-benzyl-4-oxo-3-(quinolin-4-ylmethyl)-4,5-dihydro-3H-pyrrolo[3,2d]pyrimidine-7-carbonitrile (N7F), co-crystallized with the PDB code 4A5S [14,15], available from the Protein Data Bank (PDB) [16], using the BINANA [17] to understand the molecular interaction profile of the N7F ligand. These analyses were performed to investigate the dynamics of the DPP-4 enzyme to detect important functional motions and new binding sites using the FTSite [18,19,20] and FTMap [18,20,21,22] servers.

## 2. Results and Discussion

### 2.1. Calculation of Normal Modes

**Figure 2:**
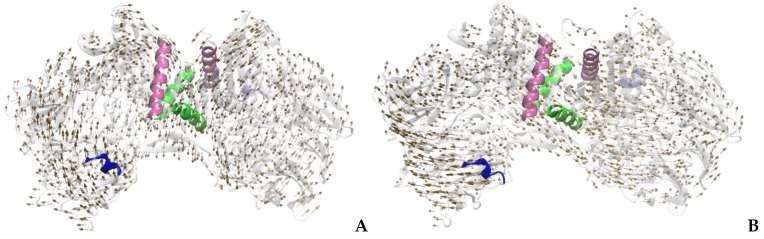
Indicative vectors of the direction of movement of the DPP-4 enzyme: (**A**) this figure corresponds to mode 7 (twisting motion between the chains); (**B**) this figure corresponds to mode 8 (active site exposure). The pink region is formed by Glu91, Asn92, Ser93, Thr94, Phe95, Asp96 and Glu97; blue is: Ser745, Thr746, Ala747, His748, Gln749, His750, Ile751, Tyr752, Thr753, His754, Met755, Ser756, His757, Phe758, Ile759, Lys760, Gln761, Cys762 and Phe763; and the green region is formed by: Phe713, Gln714, Gln715, Ser716, Ala717, Gln718, Ile719, Ser720, Lys721, Ala722, Leu723, Val724, Asp725 and Val726.

The analysis of the normal modes allowed observation of two important functional movements of the DPP-4 enzyme: a torsion (twist) and an opening motion that exposes the active site, both movements being present in all systems (with or without an inhibitor). It was found that some residues located at certain regions are involved in the movement of exposure of the active site, the β chain region (Glu91, Asn92, Ser93, Phe95, Asp96 and Glu97) and α-helix region (Ser745, Thr746, Ala747, His748, Gln749, His750, Ile751, Tyr752, Thr753, His754, Met755, Ser756, His757, Phe758, Ile759, LYS760, Gln761, Cys762 and Phe763), while in the torsion movement between the chains, in addition to the regions already mentioned, there is still the α-helix region formed by Phe713, Gln714, Gln715, Ser716, Ala717, Gln718, Ile719, Ser720, Lys721, Ala722, Leu723, Val724, Asp725, and Val726. The vectors indicative of torsional motions between the chains and the active exposure of the sites are shown in Figure 2.

**Figure 3:**
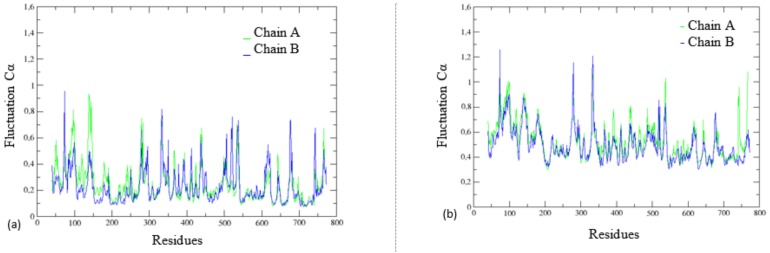

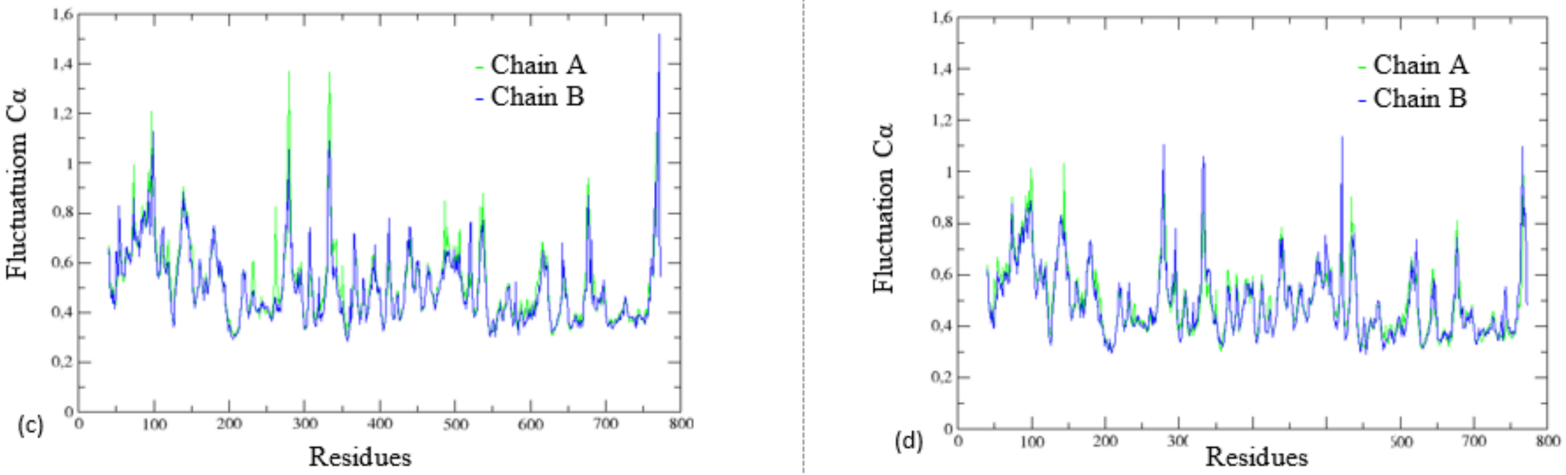
Fluctuation of α-carbon from DPP-4 (PDB: 4A5S [20,21]) at the presence and absence of inhibitor in the chains, with overlap of the two DPP-4 chains for the following systems: (**a**) dimer with inhibitor only in the chain A; (**b**) dimer with inhibitor only in the chain B; (**c**) dimer with inhibitor in both chains; (**d**) dimer without inhibitor.

By analyzing the overlapping graphs of Cα atom fluctuations of the chains A and B (Figure 3), we detected slight differences in the profile of flexibility between the DPP-4 enzyme chains, regardless of the presence of the N7F inhibitor. It was noted that there are differences in the magnitudes of the peaks of flexibility between the two DPP-4 chains, and this characteristic has not been reported in any work so far. In order to understand this phenomenon, we used several tools such as BINANA [17], FTSite [18,19,20] and FTMap [18,20,21,22].

### 2.2. Interactions between DPP-4 and the Inhibitor N7F

We used BINANA [17] to analyze the main molecular interactions of the N7F inhibitor with the DPP-4 enzyme, comparing them when the ligand is in the A chain or in the B one. The results obtained are shown in Table 1.

In the A chain of DPP-4, we found a greater number of hydrogen bond interactions than in the B chain, with the presence of π-π T-shaped-like (PIT-like )interactions only in the A chain. On the other hand, the hydrophobic contacts, π-π stacking (PIS) interactions and salt bridges were more numerous in the B chain. Thus, it can be speculated that new inhibitors have to contain functional groups that will allow for establishing interactions with a similar profile, since they are supposed to be coupled in both DPP-4 active sites (chains A and B).

We used the BINANA [17] tool to analyze the molecular interactions of 84 crystallographic structures of DPP-4 (human) enzyme with their respective ligands to verify if there were other cases in which a given ligand established different interactions in the A and B chains of DPP-4. We found that, in some cases, the biological target/ligand interaction type was different between the DPP-4 chains (these results can be seen in Table S1, Supporting Information).

### 2.3. Search for a Binding Site and Druggable Regions with FTSite and FTMap

The structure of DPP-4 (PDB code: 4A5S [14,15]) was submitted to the online servers FTSite [18,19,20] and FTMap [18,20,21,22] to locate possible binding sites and to detect their main characteristics. Considering this 3D structure (4A5S [14,15]), three binding site candidates were located at chain A, with the following residues: in site 1, Arg125, His126, Glu205, Glu206, Ser209, Phe357, Arg358, Tyr547, Ser630, Tyr631, Val656, Trp659, Tyr662, Asp663, Tyr666, Arg669, Asn710, Val711, and His740; in site 2: Arg125, Asp545, Val546, Tyr547, Lys554, Trp627, Gly628, Trp629, Ser630, Tyr631, Gly632, and His 740; and in site 3: Gly355, Arg356, Phe357, Arg358, Pro359, Ser360, Glu361, Ile374, Arg382, and Ile405.

In chain B, three similar sites were predicted with the addition of Gly549 at site 1. Residues such as Arg125, Phe357, Arg358, Tyr547, Ser630, Tyr631, and His740 were detected as an integral part of more than one binding site. Most of the residues detected at sites 1 and 2 have already been described in the literature as being present in the region of the active site [5,6,7,8,9,10,11]. The third site does not correspond to the non-catalytic sites of DPP-4 described in the literature, that promote intermolecular interactions with adenosine deaminase complexing protein 2 (ADA) (composed of Asn281, Leu294, Leu340, Val341, Ala342, and Arg343) [12,13].

**Figure 4:**
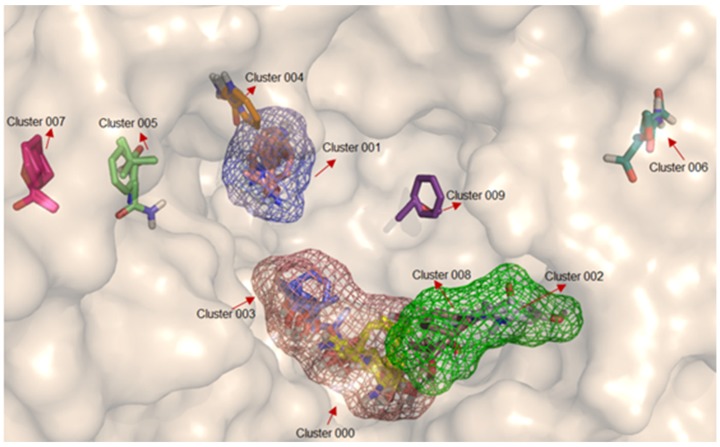
Clusters containing the molecule probes anchored in the A-chain of DPP-4 (PDB 4A5S). Site 1 in salmon, site 2 in green and site 3 in blue.

Analyzing the results obtained from FTSite and FTMap, using the standard probe molecules (mentioned in the methodology), it was possible to determine those that present the greatest affinity for the different binding sites, as follows: (1) in site 1, 16 probes (ACD, ACN, ADY, AMN, BDY, BEN, BUT, CHX, DFO, DME, EOL, ETH, PHN, THS, and URE–see definition in Table 2) present in two clusters (000 and 003), have a favorable affinity. These results indicate the affinity of polar molecules to site 1, with hydrogen bond donors and acceptors, as well as positive, hydrophobic and aromatic groups; (2) in site 2, 13 types of probes (ACN, ACD, ACT, AMN, BDY, BEN, CHX, DFO, DME, BUT, EOL, ETH, and PHN) present in clusters 002 and 008, display favorable affinities. These results indicate the same preference of this site by polar molecules; (3) in site 3, 12 types of probes (ACD, ACN, ACT, ADY, BDY, BEN, CHX, DFO, DME, ETH, PHN, and URE) present in clusters 001 and 004, indicate that this site did not show affinity with molecules with positive groups. The representation of the detected binding sites containing the coupling of the probe molecules (in clusters) according to the molecular interaction affinity in protein is illustrated in Figure 4.

The results obtained with FTMap [18,20,21,22] indicated some residues with greater contributions to hydrogen bond and nonbonded interactions, among these are Glu205, Glu206, Arg356, Phe357, Arg358, Lys554, Trp629, Tyr631, Tyr662, and Tyr666.

## 3. Materials and Methods

### 3.1. Protocol Overview-Calculation of Normal Modes (NM)

The calculation of normal modes makes it possible to investigate the large amplitude motions around an equilibrium structure [23,24,25,26]. In the case of the DPP-4 enzyme, a large system (containing two chains with more than 700 residues each), it was necessary to follow various procedures prior to the calculation of the normal modes: (1) selection of the crystallographic structure: PDB code 4A5S [14,15] with an atomic resolution of 1.62 Å (the same 3D structure that has been used in previous studies [6]), that has a structurally interesting bound ligand (with a simultaneous affinity towards the S1 and S2 sites); (2) preparation of 4 molecular systems: (a) a dimer containing an inhibitor only in the chain A of the protein; (b) a dimer containing an inhibitor only in the chain B; (c) a dimer in which both chains contain an inhibitor; (d) a dimer without an inhibitor; (3) use of CHARMM-GUI server (Quick MD Simulator) [27,28] to generate the inputs files for the minimization with GROMACS [29,30,31,32] in which we indicated the presence of disulfide bridges, an octahedral water box, the addition of 0.15M KCl ions where their positions were determined by the application of a Monte Carlo method; (4) execution of minimization with GROMACS (5.1.1) [29,30,31,32] using the above inputs; (5) the equilibrated structure obtained in the previous simulation was minimized using the CHARMM-GUI (PDB Reader) [27,33] server in order to carry out the normal mode calculations.

Firstly the systems were minimized with the conjugate gradient (CG) methods followed by the Adopted Basis Newton-Raphson algorithm (ABNR). The harmonic constraints were applied during the CG stages, progressively decreasing from 250 to 0 kcal mol^−1^ Å^−2^. Then, we minimized the systems with the unrestricted ABNR algorithm using a RMS (Root Mean Square) energy gradient convergence criterion of 10^−5^ kcal mol^−1^ Å^−1^. We calculated 81 modes using the CHARMM 40b1 [34] program with CHARMM36 [35] force field; the two lowest frequency modes corresponded to movements of torsion between the chains and the opening that exposes the active site, respectively.

### 3.2. Protocol Overview-Interactions Between DPP-4 and the N7F Inhibitor

The algorithm BINANA [17] 1.2.0 identifies the main molecular interactions established between ligands and biological targets (receptors); among them are hydrogen bonds (HB); hydrophobic contacts (HC); salt bridges (SB); π-π stacking (PIS), π-π T-stacking (PIT) and cation-π (CPI).

The ligand and receptor files were prepared in PDBQT format by adding hydrogens and Gasteiger partial charges through the tools of AutoDockTools [36]; the calculation of molecular interactions was performed using the GROMACS [29,30,31,32] equilibrated structure.

The cutoff radius values adopted correspond to the default values for each interaction: (i) π-π interaction distance cutoff: 7.5Å; (ii) cation-π distance cutoff: 6.0Å; (iii) close contacts distance cutoff: 2.5Å; (iv) active site flexibility distance cutoff: 4.0Å; (v) π-padding distance: 0.75Å; (vi) hydrophobic distance cutoff: 4.0 Å; (vii) T-stacking closest distance cutoff: 5.0Å; (viii) T-stacking angle tolerance: 30.0 Å; (ix) close contacts distance cutoff: 4.0Å; (x) π-stacking angle tolerance: 30.0Å; (xi) hydrogen bond angle cutoff: 40.0Å; (xii) hydrogen bond distance cutoff: 4.0Å; (xiii) salt bridge distance cutoff: 5.5Å; and (xiv) electrostatic distance cutoff: 4.0Å.

### 3.3. Protocol Overview-Search for Binding Sites FTSite and FTMap

In the search for possible binding sites of DPP-4, we used FTSite [18,19,20] and FTMap [18,20,21,22]. Such tools map the protein to 16 organic molecules called probes. Using the FTSite server it is possible to identify regions of possible protein binding sites, whereas FTMap [18,20,21,22] allows the characterization of the affinity profile of these regions according to the molecules-probe used. In the FTSite tool each probe is particularly placed on a dense grid around the protein. Each cluster region of the probe (cluster) is classified based on the average energy, and the consensus regions are identified as sites where different groups of probes overlap, suggesting a possible favorable region for coupling molecules. The main stages involved in FTMap [18,20,21,22] include: (i) processing of the PDB file, where ligands and water molecules are excluded; (ii) pre-docking minimization: addition of polar hydrogen atoms; (iii) Poisson-Boltzmann (PB) potential calculation: uses the CHARMM23 program to calculate PB potential around the protein; (iv) clustering of the probes and minimization, with generation of the consensus regions; (v) calculation of nonbonded interactions and hydrogen bonding (H-bonded) between the probes and the protein. The main characteristics of the probe molecules employed in both tools are shown in Table 2.

## 4. Conclusions

In this study, we observed a small difference in the flexibility profiles of the two DPP-4 chains, suggesting that chain A is more flexible and that there are fewer interactions between chain B and the inhibitors. The most significant movements observed involve the exposure of the active site and the twist between the two chains. Considering the N7F inhibitor, we detected a greater number of interactions of the hydrogen bonding and T-stacking types in the A chain of the DPP-4 enzyme.

Regarding the three sites identified here, it was noted that with the exception of the residues Phe357 and Arg358, the other residues detected at the third binding site have not yet been exploited for identification of new bioactive ligands, noting that the third site did not correspond to the non-catalytic binding sites of DPP-4 (residues Asn281, Leu294, Leu340, Val341, Ala342 and Arg343). Therefore, it might be possible that the region of the third binding site could be occupied without altering its non-catalytic functions.

In addition to structural information on the enzyme, our study contributes to understand protein dynamics in order to use this enzyme for drug design more efficiently. We proposed that the design of new compounds sharing physicochemical characteristics similar to the 16 probe molecules can be a good approach to plan efficient inhibitors of DPP-4. For site 2, it was observed that compounds containing groups with a physicochemical profile similar to the probe molecules related to acetaldehyde, isopropanol and urea were not the best compositional options for coupling in this region. In the case of site 3, molecules with structural similarity containing ethanol, isopropanol, methanamide, tert-butanol would not be suitable. The binding site 3 (see Figure 1) should be additionally studied as a new allosteric binding site. A better understanding of the functional motions of DPP-4, as well as the characteristics of the residues that compose the binding sites of this enzyme can contribute to the design of more effective DPP-4 inhibitors and further studies on side effects.

## Figures and Tables

**Table 1:** Molecular interactions detected by BINANA for the N7F inhibitor at DPP-4 (PDB code: 4A5S).

Residue	Interaction in Chain A	Interaction in Chain B
Glu205	HB/SB	SB
Phe357	-	HC
Val546	HC	-
Tyr547	HC	HC/PIS
Lys554	-	HC
Trp627	HC	HC
Gly628	HC	HC
Trp629	HC/PIS	HC
Ser630	HC	HC
Tyr631	HB	HB/HC
Val656	-	HC
Tyr662	HC	HC/PIS
Asp663	-	SB
Tyr666	HC/PIT	HC
Val711	HB	-

HC: hydrophobic contact; PIT: π-π T-shaped; PIS: π-π stacking; HB: Hydrogen bridge; SB: salt bridge; CPI: cation-π

**Table 2:** Main characteristics of the probe molecules used by FTSite and FTMap.

Molecule-probe	Physicochemical Characteristics
acetamide (ACD)	polar; donor and acceptor of hydrogen bond
acetonitrile (ACN)	polar; hydrogen acceptor binding character
acetone (ACT)	polar; hydrogen acceptor binding character
acetaldehyde (ADY)	polar; hydrogen acceptor binding character
methanamine (AMN)	polar; positive; donor and acceptor of hydrogen bond
benzaldehyde (BDY)	polar; aromatic; hydrogen acceptor binding character
benzene (BEN)	hydrophobic; aromatic
tert-butanol (BUT)	hydrophobic; aromatic
cyclohexane (CHX)	polar; donor and acceptor of hydrogen bond
*N*,*N*-dimethylformamide (DFO)	polar; hydrogen bond acceptor
dimethyl ether (DME)	polar; hydrogen bond acceptor
ethanol (EOL)	polar; donor and acceptor of hydrogen bond
ethane (ETH)	hydrophobic
phenol (PHN)	polar; aromatic; donor and acceptor of hydrogen bond
isopropanol (THS)	polar; hydrogen acceptor binding character
urea (URE)	polar; positive; donor and acceptor of hydrogen bond

## Supplementary Materials

The Supplementary Materials are available online.
