# Supplementary material for: Structural Dynamics of DPP-4 and Its Influence on the Projection of Bioactive Ligands
Source: Molecules. 2018 Feb 23;23(2):490. doi: 10.3390/molecules23020490 (PMC6017819; doi:10.3390/molecules23020490)
Supplement: Supplementary file 1 [file molecules-23-00490-s001.docx]

***Supporting Information***

Article

**Structural Dynamics of DPP-4 and Its Influence on the Projection of Bioactive Ligands**

Simone Queiroz Pantaleão ^1^, Eric Allison Philot ^2^, Pedro Túlio de Resende-Lara ^1,3^, Angélica Nakagawa Lima ^1^, David Perahia ^3^, Maria Atanassova Miteva ^4^, Ana Ligia Scott ^2,5^, Kathia Maria Honorio ^1,6^*

^1^ Center for Natural and Human Sciences, Federal University of ABC, 09210-170 Santo André, SP, Brazil; simone.queiroz@ufabc.edu.br (S.Q.P); pedro.lara@ufabc.edu.br (P.T. de R.L.); angelica.lima@ufabc.edu.br (A.N.L.)

^2^ Center for Mathematics, Computing, and Cognition, Federal University of ABC, 09210-170 Santo André, SP, Brazil; ericphilot@gmail.com (E.A.P.); analigiascott@gmail.com (A.L.S.)

^3^ École Normale Supérieure Paris-Saclay, Laboratory of Biology and Applied Pharmacology, 94235 Cachan, France; david.perahia@ens-cachan.fr

^4^ Inserm UMR-S 973-Paris Diderot University, Therapeutic Molecules by in silico approaches, 75013 Paris, France; maria.miteva@paris7.jussieu.fr

^5^ Department of Computational & Systems Biology School of Medicine, University of Pittsburgh, 15260 Pittsburgh, PA, USA; analigiascott@gmail.com (A.L.S.)

^6^ School of Arts, Sciences and Humanities, University of Sao Paulo, 03828-0000 Sao Paulo, SP, Brazil

***** Correspondence: kmhonorio@usp.br; Tel.: +55-11-3091-8856

**Table 1:** Molecular interactions detected by BINANA^[23]^ for DPP-4 inhibitors whose structures are available at PDB^[22]^

| **PDB** | **GLN 123** | **ARG**  **125** | **HIS**  **126** | **GLU**  **191** | **ASP**  **192** | **GLU 205** | **GLU 206** | **VAL**  **207** | **SER**  **209** | **PHE**  **240** | **LYS**  **250** | **VAL**  **252** | **ARG**  **253** | **ARG**  **356** | **PHE**  **357** | **ARG**  **358** | **VAL**  **546** | **TYR**  **547** | **SER**  **552** | **GLN**  **553** | **LYS**  **554** | **TYR**  **585** | **TRP**  **627** | **GLY**  **628** | **TRP**  **629** | **SER**  **630** | **TYR 631** | **GLY**  **632** | **VAL 656** | **TRP 659** | **TYR 662** | **ASP**  **663** | **TYR 666** | **ARG 669** | **ASN 710** | **VAL**  **711** | **HIS**  **740** | **GLY**  **741** |
| --- | --- | --- | --- | --- | --- | --- | --- | --- | --- | --- | --- | --- | --- | --- | --- | --- | --- | --- | --- | --- | --- | --- | --- | --- | --- | --- | --- | --- | --- | --- | --- | --- | --- | --- | --- | --- | --- | --- |
| **1N1M - Chain A** | - | - | - | - | - | HB/SB | SB | - | - | - | - | - | - | - | - | - | - | - | - | - | - | - | - | - | - | - | - | - | HC | - | HB/HC | - | HC/CPI | - | HB | HC | - | - |
| **1N1M - ChainB** | - | - | - | - | - | HB/SB | SB | - | - | - | - | - | - | - | - | - | - | - | - | - | - | - | - | - | - | HC | HC | - | HC | HC | HB/HC | - | HC/CPI | - | - | HC | - | - |
| **1NU8 - Chain B** | - | - | - | - | - | - | - | - | - | - | - | - | - | - | - | - | - | HC | - | - | - | - | - | - | - | HC | HC | - | HC | - | HC | - | HC | - | - | HC | HC | - |
| **1R9N - Chain A** | - | - | - | - | - | - | - | - | - | - | - | - | - | - | HC | - | - | HC | - | - | - | - | - | - | HC | HC | - | - | - | - | - | - | - | - | - | - | - | - |
| **1R9N - Chain B** | - | HB | - | - | - | HB | HB | - | - | - | - | - | - | - | - | - | - | HC | - | - | - | - | - | - | HC | HB/HC | HB | - | - | - | HB/HC | - | HC | - | HB | - | HB/HC | HC |
| **1RWQ - Chain A** | - | HC | - | - | - | HC | HB/HC | - | - | - | - | - | - | - | - | - | - | HB/HC | - | - | - | - | - | - | - | - | - | - | - | - | HC | - | HC/PIT | - | HB | - | - | - |
| **1RWQ - Chain B** | - | HC/CPI | - | - | - | HC | HC | - | - | - | - | - | - | - | - | - | - | HC | - | - | - | - | - | - | - | - | - | - | - | - | HC | - | HC/PIT | - | - | - | - | - |
| **1TKR - ChainA** | - | - | - | - | - | - | - | - | - | - | - | - | - | - | - | - | - | HB | - | - | - | - | - | - | - | HB | HB/HC | - | HC | HC | HC | - | HC | - | - | - | HC | - |
| **1TKR - Chain B** | - | - | - | - | - | - | - | - | - | - | - | - | - | - | - | - | - | - | - | - | - | - | - | - | - | - | - | - | - | HC | HC | - | HC | - | - | - | HC | - |
| **1WCY - Chain A** | - | HB | - | - | - | HB | HB/HC | - | - | - | - | - | - | - | - | - | - | HC | - | - | - | - | - | - | - | HB/HC | HB/HC | - | HC | - | HC | - | HC | - | - | - | HB/HC | - |
| **1WCY - ChainB** | - | HB | - | - | - | HB | - | - | - | - | - | - | - | - | HC | - | - | HB/HC | - | - | - | - | - | - | - | HB/HC | HB/HC | - | - | - | HC | - | HC | - | - | - | HB | - |
| **1X70**  **- Chain A** | - | - | - | - | - | HB/SB | HB/HC/SB | - | - | - | - | - | - | - | HC/PIS | - | - | PIS | - | - | - | - | - | - | - | HC | - | - | - | - | HB/HC/PIS | - | HC/PIT | - | - | HC | - | - |
| **1X70**  **- Chain B** | - | - | - | - | - | HB/SB | HB/HC/SB | - | - | - | - | - | - | - | HC/PIS/CPI | - | - | - | - | - | - | - | - | - | - | - | - | - | - | - | HB/HC/PIS | SB | HC/PIT | - | - | HC | - | - |
| **2AJL**  **- Chain A** | - | - | - | - | - | HB | HB | - | - | - | - | - | - | - | - | - | - | - | - | - | - | - | - | - | - | HB | HB | - | - | - | HB | - | - | - | - | - | - | - |
| **2AJL**  **- Chain B** | - | - | - | - | - | SB | SB | - | HC | - | - | - | - | - | HC | HC | - | - | - | - | - | - | - | - | - | HC | HC | - | - | - | HC | - | HC | - | - | - | - | - |
| **2BUB - Chain A** | - | HC | - | - | - | HB/HC/SB | HB/HC/SB | - | - | - | - | - | - | - | HC | - | - | HC | - | - | - | - | - | - | - | - | - | - | - | - | HB/HC | SB | HC/PIT | - | - | - | - | - |
| **2BUB - Chain B** | - | - | - | - | - | SB | HC/SB | - | - | - | - | - | - | - | HC | - | - | HC | - | - | - | - | - | - | - | HC | - | - | HC | - | HB/HC | - | HC/PIT | - | - | HC | - | - |
| **2FJP**  **- ChainA** | - | HC | - | - | - | HB/SB | HC/SB | - | - | - | - | - | - | - | HC/PIS/PIT | HB | - | - | - | - | - | - | - | - | - | - | - | - | HC | - | HC | SB | HC | - | - | HC | - | - |
| **2FJP**  **- Chain B** | - | HC | - | - | - | HB/SB | HC/SB | - | - | - | - | - | - | - | HC/PIT | HB | - | HC | - | - | - | - | - | - | - | - | - | - | HC | - | HB/HC | SB | HC | - | - | HC | - | - |
| **2G5P - Chain A** | - | HC | SB | - | - | HB/SB | HB/HC/SB | - | - | - | - | - | - | - | HC | - | - | - | - | - | - | - | - | - | - | HC | HB/HC | - | HC | - | HB/HC | - | HC | - | HB | HC | - | - |
| **2G5T - ChainA** | - | HC/CIP/SB | SB | - | - | SB | HC/SB | - | - | - | - | - | - | - | - | - | - | HB | - | - | - | - | - | - | - | HC | HB/HC | - | HC | HC | HB/HC | - | HC | - | HB | - | - | - |
| **2G63**  **- Chain B** | - | HB | - | - | - | HC/SB | HB/HC/SB | - | - | - | - | - | - | - | - | - | - | HC/PIS | - | - | HC | - | - | - | - | HC | HB/HC | - | HC | - | HB/HC | - | HC | - | HB | HC | - | - |
| **2HHA - Chain A** | - | - | - | - | - | SB | HB/SB | - | - | - | - | - | - | - | - | - | - | HC/PIS | - | HB | - | - | - | - | - | - | - | - | HC | - | HB/HC | SB | HC/PIT | - | HB | HC | - | - |
| **2HHA -**  **Chain B** | - | - | - | - | - | HB/SB | HB/SB | - | - | - | - | - | - | - | - | - | - | HC/PIS | HC | HB | - | - | - | - | - | - | - | - | HC | - | HC | SB | HC/PIT | - | HB | HC | - | - |
| **2I03**  **- Chain B** | - | CPI | HC/SB | - | - | - | HC | - | - | - | - | - | - | - | - | - | - | HC | - | - | - | - | - | - | - | HC | HB/HC | - | HC | - | HB/HC | - | HC | - | - | HC | - | - |
| **2I78**  **- Chain B** | - | - | - | - | - | HB/SB | HB/HC/SB | - | - | - | - | - | - | - | HC/PIS | - | - | - | - | - | - | - | - | - | - | - | - | - | - | - | HB/HC/PIS | SB | HC/PIT | - | - | HC | - | - |
| **2IIT**  **- Chain A** | - | - | - | - | - | SB | HB/HC/SB | - | - | - | - | - | - | - | HC | - | - | - | - | - | - | - | - | - | - | HC | - | - | - | - | HB/HC/PIS | SB | HC/PIT | - | - | HC | - | - |
| **2IIT**  **- Chain B** | - | - | - | - | - | SB | HB/HC/SB | - | - | - | - | - | - |  | HC | - | - | - | - | - | - | - | - | - | - | HC | - | - | - | - | HB/HC/PIS | SB | HC/PIT | - | - | HC | - | - |
| **2IIV**  **- Chain A** | - | - | - | - | - | HB/SB | HB/SB | - | - | - | - | - | - |  | HC | - | - | - | - | - | - | - | - | - | - | - | - | - | - | - | HB/HC/PIS | SB | HC/PIT | - | - | HC | - | - |
| **2IIV**  **- Chain B** | - | - | - | - | - | HB/SB | HB/HC/SB | - | - | - | - | - | - | - | HC | - | - | - | - | - | - | - | - | - | - | - | - | - | - | - | HB/HC/PIS | SB | HC/PIT | - | - | HC | - | - |
| **2JID - Chain A** | - | HC/CPI | - | - | - | HB/SB | HB/SB | - | - | - | - | - | - | - | - | - | - | - | - | - | - | - | - | - | - | HC | HC | - | HC | - | HB/PIS | SB | HC/PIT | - | - | HC | - | - |
| **2JID - Chain B** | - | HC/CPI | - | - | - | HB/SB | HB/HC/SB | - | - | - | - | - | - | - | - | - | - | PIT | - | - | - | - | - | - | - | HC | - | - | - | - | HB/HC/PIS | - | HC/PIT | - | - | HC | HC | - |
| **2OAG - Chain B** | - | - | - | - | - | HB/SB | HB/HC/SB | HC | - | - | - | - | - | - | HC/PIS | HB | - | - | - | - | - | - | - | - | - | HC | - | - | - | - | HC | SB | HC/PIT | - | HB | HC | HC | - |
| **2OGZ - Chain A** | - | - | - | - | - | HC | HC | - | - | - | - | - | - | - | HC | - | - | HC/PIS | - | - | - | - | - | - | - | HC | HB/HC | HB | HC | - | HC | - | HC/HC/PIT | - | HB | HC | - | - |
| **2OGZ - Chain B** | - | - | - | - | - | HC | HC | - | - | - | - | - | - | - | HC | - | - | HC/PIS | - | - | - | - | - | - | - | HC | HB/HC | HB/HC | HC | - | HC | - | HC/PIT | - | - | HC | - | - |
| **2OLE Chain A** | - | - | - | - | - | SB | SB | - | - | - | - | - | - | - | HC/CPI | - | - | - | - | - | - | - | - | - | - | HC | - | - | - | - | - | SB | HC | - | - | HC | - | - |
| **2OLE - Chain B** | - | - | - | - | - | SB | SB | - | - | - | - | - | - | - | HC/CPI | - | - | - | - | - | - | - | - | - | - | HC | - | - | - | - | HC | SB | HC | - | - | HC | HC | - |
| **2ONC - Chain A**  **- conformation 1** | - | - | - | - | - | HB/SB | SB | - | - | - | - | - | - | - | HC | - | - | HC/PIS | - | - | - | - | - | - | - | HC | HB/HC | - | HC | - | HC/PIS | - | HC/PIT | - | - | HC | - | - |
| **2ONC - Chain B**  **- conformation 1** | - | - | - | - | - | HB/SB | SB | - | - | - | - | - | - | - | HC | - | - | HC/PIS | - | - | - | - | - | - | - | HC | HB/HC | - | HC | - | HC/PIS | - | HC/PIT | - | - | HC | - | - |
| **2ONC - Chain A - conformation 2** | - | - | - | HC/SB | HB/HC/SB | - | - | - | - | HC/PIT | HC/CPI | HC | HB | - | - | - | - | - | - | - | - | - | - | - | - | - | - | - | - | - | - | - | - | - | - | - | - | - |
| **2ONC - Chain B**  **- conformation 2** | HB | - | - | HC | HB/HC/SB | - | - | - | - | HC/PIT | HC/CPI | HC | HB | - | - | - | - | - | - | - | - | - | - | - | - | - | - | - | - | - | - | - | - | - | - | - | - | - |
| **2OPH - ChainA** | - | - | - | - | - | SB | SB | - | - | - | - | - | - | - | HC | - | - | - | - | - | - | - | - | - | - | - | - | - | HC | - | HB/HC | - | HC | - | - | HC | - | - |
| **2OPH - Chain B** | - | - | - | - | - | SB | SB | - | - | - | - | - | - | - | HC | HB | - | - | - | - | - | - | - | - | - | - | - | - | HC | - | HC | - | HC | - | - | HC | - | - |
| **2OQI**  **- Chain B** | - | - | - | - | - | SB | HB/SB | - | HC | - | - | - | - | - | HC/PIS | - | - | - | - | - | - | - | - | - | - | HC | - | - | - | - | HB/HC/PIS | - | HC/PIT | - | - | HC | - | - |
| **2OQV - Chain A** | - | - | - | - | - | HB/SB | HB/SB | HC | - | - | - | - | - | - | HC/PIS | HC | - | - | - | - | - | - | - | - | - | HC | - | - | - | - | HC | - | HC/PIT | - | - | HC | - | - |
| **2P8S - Chain A**  **-**  **conformation 1** | - | - | - | - | - | HB/SB | HB/SB |  | HC | - | - | - | - | - | CPI | - | - | - | - | - | - | - | - | - | - | - | - | - | - | - | HB/HC/PIS | SB | HC/PIT | - | - | HC | - | - |
| **2P8S**  **- Chain B**  **-**  **conformation 1** | - | - | - | - | - | HB/SB | HB/SB | - | HC | - | - | - | - | - | CPI | - | - | - | - | - | - | - | - | - | - | - | - | - | - | - | HB/HC/PIS | SB | HC/PIT | - | - | HC | - | - |
| **2P8S**  **- Chain A**  **-**  **conformation 2** | - | - | - | - | - | HB/SB | HB/SB | - | - | - | - | - | - | - | HC/PIS/CPI | - | - | - | - | - | - | - | - | - | - | - | - | - | - | - | HB/HC/PIS | SB | HC/PIT | - | - | HC | - | - |
| **2P8S**  **- Chain B**  **-**  **conformation 2** | - | - | - | - | - | HB/SB | HB/SB | - | - | - | - | - | - | - | HC/PIS/CPI | - | - | - | - | - | - | - | - | - | - | - | - | - | - | - | HB/HC/PIS | - | HC/PIT | - | HC | HC | - | - |
| **2QJR**  **- Chain A** | - | - | - | - | - | HB/SB | HB/HC/SB | - | - | - | - | - | - | - | HC/PIS | HB/HC | - | - | - | - | - | - | - | - | - | HC | - | - | - | - | HB/HC/PIS | SB | HC/PIT | - | - | HC | - | - |
| **2QJR**  **- Chain B** | - | - | - | - | - | HB/SB | HB/SB | - | - | - | - | - | - | - | HC/PIS | HB | - | - | - | - | - | - | - | - | - | - | - | - | - | - | HB/PIS | SB | HC/PIT | - | - | HC | - | - |
| **2QKY**  **- Chain A** | - | HB | - | - | - | HB | - | - | - | - | - | - | - | - | - | - | - | HB/HC | - | - | - | - | - | - | - | HB/HC | - | - | HC | - | HC | - | HC | - | - | HC | - | - |
| **2QKY**  **- Chain B** | - | HB | - | - | - | HB | HC | - | - | - | - | - | - | - | HC | - | - | HC | - | - | - | - | - | - | - | HB/HC | HB | - | - | - | HC | - | HC | - | - | HC | HC | - |
| **2QOE - Chain A** | - | - | - | - | - | HB/SB | HB/SB | - | - | - | - | - | - | - | HC/PIS | - | - | - | - | - | - | - | - | - | - | - | - | - | - | - | HB/HC/PIS | SB | HC/PIT | - | - | HC | - | - |
| **2QOE**  **- Chain B** | - | - | - | - | - | HB/SB | HB/SB | - | - | - | - | - | - | - | HC/PIS | - | - | - | - | - | - | - | - | - | - | - | - | - | - | - | HB/HC/PIS | SB | HC/PIT | - | - | HC | - | - |
| **2QT9 - Chain A** | - | HC | - | - | - | SB | SB | - | - | - | - | - | - | - | HC | HC | - | HC | - | - | - | - | - | - | - | - | - | - | - | - | HB/HC | SB | HC | - | HB | HC | - | - |
| **2QT9 - Chain B** | - | - | - | - | - | HB/SB | SB | - | - | - | - | - | - | - | HC | HC | - | HC | - | - | - | - | - | - | - | - | - | - | - | - | HB/HC | SB | HC | - | HB | HC | - | - |
| **2QTB - Chain A** | - | HC | - | - | - | HB/SB | SB | - | - | - | - | - | - | - | HC | HB/HC | - | HC | - | - | - | - | - | - | - | - | - | - | HC | - | HB/HC | SB | HC | - | HB | HC | - | - |
| **2QTB - Chain B** | - | - | - | - | - | HB/SB | SB | - | - | - | - | - | - | - | HC | HB/HC | - | HC | - | - | - | - | - | - | - | - | - | - | HC | - | HB/HC | SB | HC | - | HB | HC | - | - |
| **2RGU - Chain A** | - | - | - | - | - | SB | HB/SB | - | - | - | - | - | - |  | HC | - | - | HC/PIS | - | - | - | - | - | - | HC/PIS | - | HC | - | HC | - | - | - | HC | - | - | HC | HC | - |
| **2RGU - Chain B** | - | - | - | - | - | SB | HB/SB | - | - | - | - | - | - | - | - | HC | - | HC/PIS/PIT | - | - | - | - | - | - | HC/PIS | - | HB/HC | - | - | - | HB/HC | - | HC | - | - | HC | HC | - |
| **2RIP**  **- Chain A** | - | - | - | - | - | HB/SB | HB/HC/SB | - | - | - | - | - | - | - | HC | HC | - | - | - | - | - | - | - | - | - | - | - | - | HC | - | HB/HC/PIS | SB | HC/PIT | CPI | HB | HC | - | - |
| **3BJM - Chain A** | - | - | - | - | - | HB/SB | HB/SB | - | - | - | - | - | - | - | HC | - | - | HB | - | - | - | - | - | - | - | HC | HC | - | HC | - | HB/HC | SB | HC | - | HB | HC | - | - |
| **3BJM - Chain B** | - | - | - | - | - | HB/SB | HB/SB | - | - | - | - | - | - | - | HC | - | - | HB | - | - | - | - | - | - | - | HB/HC | HC | - | HC | - | HB/HC | SB | HC | - | HB | HC | - | - |
| **3C43 - Chain A** | - | - | - | - | - | SB | HB/SB | - | - | - | - | - | - | - | - | - | - | HC/PIS | HC | HB | - | - | - | - | - | - | - | - | HC | - | HB/HC | SB | HC | - | - | HC | - | - |
| **3C43 - Chain B** | - | - | - | - | - | SB | HB/SB | - | - | - | - | - | - | - | - | - | - | HC | - | HB | - | - | - | - | - | - | - | - | HC | - | HB/HC | SB | HC | - | HB | HC | - | - |
| **3C45 - Chain A** | - | - | - | - | - | HB/SB | HB/SB | - | - | - | - | - | - | - | - | - | - | HC/PIS | HC | HC | HC | - | - | - | - | - | - | - | HC | - | HB/HC | SB | HC | - | - | HC | - | - |
| **3C45 - Chain B** | - | - | - | - | - | HB/SB | HB/SB | - | - | - | - | - | - | - | - | - | - | HC/PIS | HC | HC | HC | - | - | - | - | - | - | - | HC | - | HB/HC/PIS | SB | HC/PIT | - | - | HC | - | - |
| **3CCB - Chain A** | - | HC/CPI | - | - | - | HB/SB | HB/HC/SB | - | - | - | - | - | - | - | - | - | - | - | - | - | - | - | - | - | - | HC | - | - | - | - | HB/HC/PIS | SB | HC/PIT | - | - | HC | - | - |
| **3CCB - Chain B** | - | HC/CPI | - | - | - | HB/SB | HB/SB | - | - | - | - | - | - | - | - | - | - | - | - | - | - | - | - | - | - | HC | - | - | HC | - | HC/PIS | SB | HC/PIT | - | - | HC | - | - |
| **3CCC - ChainA** | - | HC/CPI | - | - | - | HB/HC/SB | HB/HC/SB | - | - | - | - | - | - | - | - | - | - | HC | - | - | - | - | - | - | - | HC | HB/HC | - | HC | - | HC/PIS | - | HC/PIT | - | HB | HC | - | - |
| **3CCC - Chain B** | - | CPI | - | - | - | HB/SB | HB/HC/SB | - | - | - | - | - | - |  | PIS | - | - | - | - | - | - | - | - | - | - | HC | HB/HC | - | HC | - | HB/HC/PIS | SB | HC/PIT | - | - | HC | - | - |
| **3D4L - Chain A** | - | - | - | - | - | HB/SB | HB/SB | HB | HB | - | - | - | - | - | HC | HB | - | - | - | - | - | - | - | - | - | - | - | - | HC | - | HB/HC | - | HC/PIT | - | - | HC | - | - |
| **3D4L - Chain B** | - | - | - | - | - | HB/SB | HB/SB | HB | HB | - | - | - | - | - | HC/PIS | HB | - | - | - | - | - | - | - | - | - | - | - | - | HC | - | HB/HC/PIS | - | HC/PIT | - | - | HC | - | - |
| **3EIO - Chain A** | - | - | - | - | - | HB/SB | HB/SB | - | - | - | - | - | - | SB | HC | - | - | - | - | - | - | - | - | - | - | HC | - | - | - | - | HB/HC/PIS | SB | HC/PIT | - | - | HC | - | - |
| **3EIO - Chain B** | - | - | - | - | - | HB/SB | HB/SB | - | - | - | - | - | - | SB | HC | - | - | - | - | - | - | HB | - | - | - | HC | - | - | - | - | HB/HC/PIS | SB | HC/PIT | - | - | HC | - | - |
| **3F85 - ChainA** | - | - | - | - | - | HB/SB | HB/SB | - | - | - | - | - | - | SB | HC | - | - | - | - | - | - | - | - | - | - | HC | - | - | - | - | HB/HC/PIS | SB | HC/PIT | - | - | HC | - | - |
| **3F85 - Chain B** | - | - | - | - | - | HB | HB | - | - | - | - | - | - | - | HC | - | - | - | - | - | - | HB | - | - | - | HC | - | - | - | - | HB/HC | - | HC | - | - | HC | - | - |
| **3G0B - Chain A** | - | - | - | - | - | SB | SB | - | - | - | - | - | - | - | - | - | - | HC/PIS | - | - | - | - | - | - | - | HC | HB | - | HC | - | HC/PIS | - | HC/PIT | - | - | HC | - | - |
| **3G0B - Chain B** | - | - | - | - | - | HC/SB | SB | - | - | - | - | - | - | - | HC | - | - | HC/PIS | - | - | - | - | - | - | - | HC | HB/HC | - | HC | - | HC/PIS | - | HC/PIT | - | - | HC | - | - |
| **3G0C - ChainA** | - | - | - | - | - | HB/SB | HB/HC/SB | - | - | - | - | - | - | - | - | - | - | HC/PIS | - | - | - | - | - | - | - | HC | HB | - | - | - | HC | - | HC | - | - | HC | HC | - |
| **3G0C - Chain B** | - | - | - | - | - | SB | HB/HC/SB | - | - | - | - | - | - | - | - | - | - | HC/PIS | - | - | - | - | - | - | - | HC | HB | - | - | - | HC | - | HC/PIT | - | HC | HC | HC | - |
| **3G0D - Chain A** | - | - | - | - | - | SB | SB | - | - | - | - | - | - | - | HC | - | - | HC/PIS | - | - | - | - | - | - | - | HC | HB/HC | - | HC | - | HC/PIS | - | HC/PIT | - | - | HC | - | - |
| **3G0D - Chain B** | - | - | - | - | - | SB | SB | - | - | - | - | - | - | - | - | - | - | HC/PIS | - | - | - | - | - | - | HC | HC | HB/HC | - | HC | - | HC/PIS | - | HC/PIT | - | - | HC | - | - |
| **3G0G – Chain A** | - | - | - | - | - | SB | SB | - | - | - | - | - | - | - | - | - | - | HC | - | - | - | - | - | - | - | HC | HB/HC | - | HC | - | HC | - | HC/PIT | - | - | HC | - | - |
| **3G0G - Chain B** | - | - | - | - | - | SB | HB/SB | - | - | - | - | - | - | - | - | - | - | HC/PIS | - | - | - | - | - | - | - | HC | HB | - | HC | - | HC/PIS | - | HC/PIT | - | - | HC | - | - |
| **3H0C - Chain A** | - | - | - | - | - | HB/SB | HB/SB | - | - | - | - | - | - | - | HC | - | - | HC/HC/PIS | HC | HB | HC | - | - | - | - | - | - | - | - | - | HB/HC/PIS | SB | HC/PIT | - | HC | HC | - | - |
| **3H0C - Chain B** | - | - | - | - | - | HB/SB | HB/SB | - | - | - | - | - | - | - | HC | - | - | HC/PIS | HC | HB | - | - | - | - | - | - | - | - | - | - | HB/HC | SB | HC/PIT | - | - | HC | - | - |
| **3HAB - Chain A** | - | - | - | - | - | HB/SB | HB/HC/SB | - | - | - | - | - | - | - | HC/PIS | - | - | - | - | - | - | - | - | - | - | - | - | - | - | - | HB/HC/PIS | SB | HC/PIT | - | - | HC | - | - |
| **3HAB**  **- Chain B** | - | - | - | - | - | HB/SB | HB/HC/SB | - | - | - | - | - | - | - | HC/PIS | - | - | - | - | - | - | - | - | - | - | - | - | - | - | - | HB/HC/PIS | SB | HC/PIT | - | - | HC | - | - |
| **3HAC - ChainA** | - | - | - | - | - | HB/SB | HB/HC/SB | - | - | - | - | - | - | - | HC/PIS | - | - | - | - | - | - | - | - | - | - | - | - | - | - | - | HB/HC/PIS | SB | HC/PIT | - | - | HC | - | - |
| **3HAC - Chain B** | - | - | - | - | - | HB/SB | HB/HC/SB | - | - | - | - | - | - | - | HC/PIS | - | - | - | - | - | - | - | - | - | - | - | - | - | - | - | HB/HC/PIS | SB | HC/PIT | - | - | HC | - | - |
| **3KWF - Chain A** | - | - | - | - | - | HB/SB | HB/HC/SB | - | - | - | - | - | - | - | HC/PIS | - | - | - | - | - | - | - | - | - | - | HC | HC | - | - | - | HB/HC | - | HC | - | HB | HC | - | - |
| **3KWF - Chain B** | - | - | - | - | - | HB | HB/HC/SB | SB | - | - | - | - | - | - | HC/PIS | - | - | - | - | - | - | - | - | - | - | - | HC | - | HC | - | HB/HC | SB | HC | - | HC | - | - | - |
| **3KWJ - ChainA** | - | - | - | - | - | - | HB/SB | HB/SB | - | - | - | - | - | - | HC | - | - | - | - | - | - | - | - | - | - | - | HC | - | HC | HC | HB/HC/PIS | - | HC/PIT | - | - | HC | - | - |
| **3KWJ - Chain B** | - | - | - | - | - | - | HB/SB | HB/HC/SB | - | - | - | - | - | - | HC | - | - | - | - | - | - | - | - | - | - | - | HC | - | - | - | HB/HC/PIS | - | HC/PIT | - | - | HC | HC | - |
| **3NOX - Chain A** | - | HC/CPI | - | - | - | - | HB/SB | HB/HC/SB | - | - | - | - | - | - | - | - | - | HC | - | - | - | - | - | - | - | HC | HC | - | - | - | HC/PIS | - | HC/PIT | - | - | - | - | - |
| **3NOX - Chain B** | - | CPI | - | - | - | - | HC | - | - | - | - | - | - | - | - | - | - | HC | - | - | - | - | - | - | HC | HC | - | - | - | - | HB/HC/PIS | - | HC/PIT | - | - | - | - | - |
| **3O9V - Chain A** | - | HB/HC/CPI/SB | - | - | - | - | HB/SB | HB/HC/SB | - | - | - | - | - | - | - | - | - | HB | - | - | - | - | - | - | - | HB | - | - | - | - | HC/PIS | SB | HC/PIT | - | - | HC | SB | - |
| **3O9V - Chain B** | - | HB/HC/CPI | - | - | - | - | SB | HB/HC/SB | - | - | - | - | - | - | - | - | - | HB | - | - | - | - | - | - | - | HB | - | - | - | - | HC/PIS | SB | HC/PIT | - | - | HC | SB | - |
| **3O95 – Chain A** | - | HB/HC/CPI/SB | - | - | - | - | HB/SB | HB/HC/SB | - | - | - | - | - | - | HC/PIS | - |  | - | - | - | - | - | - | - | - | - | - | - | HC | - | HC/PIS | SB | HC/PIT | - | - | HC | HC/SB | - |
| **3O95 - Chain B** | - | HB/HC/CPI | - | - | - | HB/SB | HB/HC/SB | - | - | - | - | - | - | - | HC/PIS | - | - | - | - | - | - | - | - | - | - | HB | - | - | HC | - | HB/HC | SB | HC/PIT | - | - | - | HC/SB | - |
| **3OC0 - Chain A** | - | - | - | - | - | SB | HC/SB | - | - | - | - | - | - | - | HC/PIS | HB | - | - | - | - | - | - | - | - | - | - | - | - | HC | HC | HB/HC | - | HC | - | - | - | - | - |
| **3OC0 - Chain B** | - | - | - | - | - | HB/SB | HC/SB | - | - | - | - | - | - | - | HC/PIS | - | - | - | - | - | - | - | - | - | - | - | - | - | - | - | HB | - | HC | - | - | HC | - | - |
| **3OPM - Chain A** | - | CPI | - | - | - | HB/SB | HB/HC/SB | - | - | - | - | - | - | - | HC/PIS | - | - | HC/PIS | - | - | - | - | - | - | - | HC | HC | - | - | - | HC/PIS | SB | HC/PIT | - | - | HC | - | - |
| **3OPM - Chain B** | - | CPI | - | - | - | SB | HB/HC/SB | - | - | - | - | - | - | - | HC/PIS | - | - | HC/PIS | - | - | - | - | - | - | - | - | HC | - | - | - | HC/PIS | SB | HC | - | - | HC | - | - |
| **3Q0T - Chain A** | - | HC | - | - | - | HB/SB | HB/HC/SB | - | - | - | - | - | - | - | - | - | - | HC | - | - | - | - | - | - | - | - | HC | - | - | - | HC/PIS | SB | HC | - | - | - | - | - |
| **3Q0T - Chain B** | - | HC/CPI | - | - | HB | HB/SB | HC/SB | - | - | - | - | - | - | - | - | - | - | HC | - | - | - | - | - | - | - | - | - | - | - | - | HB/HC/PIS | SB | HC/PIT | - | - | HC | - | - |
| **3Q8W - Chain A** | - | HB/SB | - | - | - | HB/SB | HB/SB | - | - | - | - | - | - | - | HC | - | - | HC | - | - | - | - | - | - | HC | HC | - | - | - | - | HC/PIS | - | PIT | - | - | HC | SB | - |
| **3Q8W - Chain B** | - | HB/SB | - | - | - | HB/SB | HB/HC/SB | - | - | - | - | - | - | - | HC | - | - | HC | - | - | - | - | - | - | HC | - | HC | - | - | - | - | - | PIT | - | - | - | SB | - |
| **3QBJ - ChainA** | - | - | - | - | - | HB/SB | HB/HC/SB | - | - | - | - | - | - | - | HC/PIS | HC | - | - | - | - | - | - | - | - | - | HC | - | - | - | - | HB/HC | SB | HC | - | HB | HC | - | - |
| **3QBJ - Chain B** | - | - | - | - | - | HB/SB | HB/HC/SB | - | - | - | - | - | - | - | HC/PIS | HC | - | - | - | - | - | - | - | - | - | - | - | - | - | - | HB/HC | SB | HC | - | HB | HC | - | - |
| **3SWW - Chain A** | - | HC/CPI | - | - | - | HB/SB | HB/HC/SB | - | - | - | - | - | - | - | - | - | - | - | - | - | - | - | - | - | HC | - | - | - | - | - | HC/PIS | SB | HC/PIT | - | - | HC | - | - |
| **3SWW - Chain B** | - | HC/CPI | - | - | - | HB/SB | HB/HC/SB | - | - | - | - | - | - | - | - | - | - | - | - | - | - | - | - | - | HC | - | - | - | - | - | HB/HC/PIS | SB | HC/PIT | - | - | HC | - | - |
| **3SX4**  **- Chain A -**  **conformation 1** | - | HC | - | - | - | HC/SB | HB/HC/SB | - | - | - | - | - | - | - | - | - | - | HC/PIS | - | - | - | - | - | - | - | - | HC | - | - | - | HC | SB | HC/PIT | - | - | - | - | - |
| **3SX4**  **- ChainB - conformation 1** | - | - | - | - | - | SB | HB/HC/SB | - | - | - | - | - | - | - | - | - | - | HC | - | - | - | - | - | - | HC | HC | - | - | - | - | HC/PIS | SB | HC/PIT | - | - | - | - | - |
| **3SX4**  **- Chain A -**  **conformation 2** | - | HB/HC | - | - | - | SB | SB | - | - | - | - | - | - | - | - | - | - | - | - | - | - | - | - | - | HC/PIS | HC | HC | - | - | - | HC | - | HC | - | - | HC | HC | HC |
| **3SX4**  **- ChainB -**  **conformation 2** | - | HB/HC | - | - | - | HB/SB | HB/SB | - | - | - | - | - | - | - | - | - | - | HC | - | - | - | - | - | - | HC/PIS | HC | HC | - | - | - | HB/HC/PIS | SB | HC | - | - | - | HC | HC |
| **3VJK**  **- ChainA** | - | - | - | - | - | HB/HC/SB | HB/HC/SB | - | HC | - | - | - | - | - | HC/PIS | HC | - | - | - | - | - | - | - | - | - | - | HC | - | - | HC | HB | SB | HC | - | HB | HC | - | - |
| **3VJK**  **- Chain B** | - | - | - | - | - | HB/HC/SB | HB/HC/SB | HC | HC | - | - | - | - | - | HC/PIS | HC | - | - | - | - | - | - | - | - | - | - | - | - | - | - | HC | - | HC | - | HB | HC | - | - |
| **3VJL**  **- Chain A** | - | - | - | - | - | HB/HC/SB | HB/HC/SB | - | - | - | - | - | - | - | HC | HC | - | - | - | - | - | - | - | - | - | - | - | - | - | - | HC | SB | HC | - | HB | HC | - | - |
| **3VJL**  **- Chain B** | - | - | - | - | - | HB/HC/SB | HB/HC/SB | - | - | - | - | - | - | - | HC | - | - | - | - | - | - | - | - | - | - | HC | - | - | - | - | HC | - | HC/PIT | - | HB | HC | - | - |
| **3VJM**  **- Chain A** | - | - | - | - | - | HB/HC/SB | HB/HC/SB | - | - | - | - | - | - | - | HC/PIS | HC | - | - | - | - | - | - | - | - | - | - | HC | - | - | HC | HB/HC | SB | HC/HC | - | HB | HC | - | - |
| **3VJM**  **- Chain B** | - | - | - | - | - | HB/SB | HB/SB | - | - | - | - | - | - | - | HC/PIS | HC | - | - | - | - | - | - | - | - | - | - | - | - | - | HC | HC | SB | HC | - | HB | - | - | - |
| **3WQH**  **- Chain A** | - | - | - | - | - | HB | HC | - | - | - | - | - | - | - | HC/PIS | - | - | HB | - | - | - | - | - | - | - | HC | - | - | - | - | HC | - | HC | - | - | - | HB | - |
| **3WQH**  **- Chain B** | - | - | - | - | - | HC | HC | - | - | - | - | - | - | - | HC/PIS | HB | - | - | - | - | - | - | - | - | - | HC | - | - | - | - | HC | - | HC | - | - | - | HB | - |
| **4DSA**  **- Chain A** | - | - | - | - | - | SB | HC/SB | - | - | - | - | - | - | - | HC/PIS | - | - | - | - | - | - | - | - | - | - | - | - | - | - | - | HB/HC | - | HC/PIT | - | - | HC | - | - |
| **4DSZ**  **- Chain A** | - | - | - | - | - | HB/SB | HB/SB | - | - | - | - | - | - | - | HC/PIS | - | - | - | - | - | - | - | - | - | - | - | - | - | - | - | HC/PIS | - | HC | - | - | HC | - | - |
| **4DSZ**  **- Chain B** | - | - | - | - | - | HB/SB | HC/SB | - | - | - | - | - | - | - | HC/PIS | - | - | - | - | - | - | - | - | - | - | - | - | - | - | - | HB/HC/PIS | - | HC/PIT | - | - | HC | - | - |
| **4DTC**  **- Chain A** | - | - | - | - | - | HB/SB | HB/SB | - | - | - | - | - | - | - | HC/PIS | HC | - | - | - | - | - | - | - | - | - | - | - | - | - | - | HB/HC/PIS | - | HC/PIT | - | - | HC | - | - |
| **4DTC**  **- Chain B** | - | - | - | - | - | HB/SB | HC/SB | - | - | - | - | - | - | - | HC/PIS | HC | - | - | - | - | - | - | - | - | - | - | - | - | - | - | HB/HC/PIS | SB | HC/PIT | - | - | HC | - | - |
| **4GIF**  **- Chain A** | - | - | - | - | - | SB | HB/HC/SB | - | - | - | - | - | - | - | - | - | - | HC/PIS | - | - | - | - | - | - | - | HC | HB/HC | - | HC | - | HB/HC/PIS | SB | HC/PIT | - | - | HC | - | - |
| **4GIF**  **- Chain B** | - | - | - | - | - | SB | HB/HC/SB | - | - | - | - | - | - | - | - | - | - | HC/PIS | - | - | - | - | - | - | - | HC | HB/HC | - | HC | - | HC/PIS | SB | HC/PIT/CPI | - | - | HC | - | - |
| **4J3J**  **- Chain A** | - | - | - | - | - | HB/SB | HB/SB | - | - | - | - | - | - | - | HC | - | - | - | - | - | - | - | - | - | - | - | - | - | - | - | HB/HC/PIS | - | HC/PIT | - | - | HC | - | - |
| **4J3J**  **- Chain B** | - | - | - | - | - | SB | SB | - | - | - | - | - | - | - | HC | - | - | - | - | - | - | - | - | - | - | - | - | - | - | - | HB/HC/PIS | - | HC/PIT | - | - | HC | - | - |
| **4JH0**  **- Cadeia A** | - | HC/CPI | - | - | - | HB/HC/SB | HB/HC/SB | - | - | - | - | - | - | - | - | - | - | HB/HC | - | - | - | - | - | - | - | HB | - | - | - | - | HC/PIS | SB | HC/PIT | - | - | - | - | - |
| **4JH0**  **- Chain B** | - | CPI | - | - | - | HB/HC/SB | HB/HC/SB | - | - | - | - | - | - | - | - | - | - | HC | - | - | - | - | - | - | - | HB | - | - | - | - | HB/HC/PIS | SB | HC/PIT | - | - | - | - | - |
| **4LKO Cadeia A** | - | - | - | - | - | HB/HC/SB | HB/HC/SB | - | - | - | - | - | - | - | - | - | - | HC | - | - | - | - | - | - | - | HB | - | - | - | - | HB/HC/PIS | SB | HC/PIT | - | - | HC | - | - |
| **4LKO**  **- Chain B** | - | - | - | - | - | HB/SB | HB/HC/SB | - | - | - | - | - | - | - | - | - | - | HC | - | - | - | - | - | - | - | HB | - | - | - | - | HC/PIS | SB | HC/PIT | - | - | - | - | - |
| **4N8D - Chain A** | - | - | - | - | - | HB/SB | HB/HC/SB | - | HC | - | - | - | - | - | HC | CPI | - | - | - | - | - | - | - | - | - | HC | - | - | - | - | HB/HC/PIS | SB | HC/PIT | - | - | HC | - | - |
| **4N8D - Chain B** | - | - | - | - | - | HB/SB | HB/HC/SB | - | - | - | - | - | - | - | HC | HC/CPI | - | - | - | - | - | - | - | - | - | HC | - | - | HC | - | HB/HC/PIS | SB | HC/PIT | - | - | HC | - | - |
| **4N8E - Chain A** | - | - | - | - | - | HB/SB | HB/SB | - | - | - | - | - | - | - | HC | - | - | - | - | - | - | - | - | - | - | HC | - | - | - | - | HB/HC/PIS | SB | HC/PIT | - | - | HC | - | - |
| **4N8E - Chain B** | - | - | - | - | - | HB/SB | HB/HC/SB | - | - | - | - | - | - | - | HC | - | - | - | - | - | - | - | - | - | - | HC | - | - | - | - | HB/HC/PIS | SB | HC/PIT | - | - | HC | - | - |
| **4PNZ - Chain A** | - | - | - | - | - | HB/SB | HB/SB | - | - | - | - | - | - | - | HC/PIS/CPI | - | - | - | - | - | - | - | - | - | - | - | - | - | - | - | HB/HC/PIS | SB | HC/PIT | - | - | HC | - | - |
| **4PNZ - Chain B** | - | - | - | - | - | HB/SB | HB/SB | - | - | - | - | - | - | - | HC/PIS/CPI | - | - | - | - | - | - | - | - | - | - | - | - | - | - | - | HB/HC/PIS | SB | HC/PIT | - | - | HC | - | - |
| **4PV7**  **- Chain A** | - | HC | - | - | - | HC | HB/HC | - | - | - | - | - | - | - | - | - | - | - | - | - | - | - | - | - | - | HC | - | - | - | - | HC | - | HC/HC | - | - | - | - | - |
| **4PV7**  **- Chain B** | - | HC | - | - | - | HC | HB/HC | - | - | - | - | - | - | - | - | - | - | - | - | - | - | - | - | - | - | HC | - | - | - | - | - | - | HC | - | - | - | - | - |
| **5I7U**  **- Chain A** | - | - | - | - | - | SB | SB | - | - | - | - | - | - | - | HC | - | - | HC/PIS | - | - | - | - | - | - | - | HC | HB/HC | - | HC | - | HC/PIS | - | HC/PIT | - | - | HC | - | - |
| **5I7U**  **- Chain B** | - | - | - | - | - | SB | SB | - | - | - | - | - | - | - | HC | - | - | HC/PIS | - | - | - | - | - | - | - | HC | HB/HC | - | HC | - | HC/PIS | - | HC/PIT | - | - | HC | - | - |
| **5ISM - Chain A** | - | - | - | - | - | HB/SB | HB/SB | - | - | - | - | - | - | - | HC/PIS | - | - | - | - | - | - | - | - | - | - | - | - | - | HC | - | HB/HC/PIS | SB | HC/PITR | - | - | HC | - | - |
| **5ISM**  **- Chain B -**  **conformation 1** | - | - | - | - | - | HB/SB | HB/SB | - | - | - | - | - | - | - | HC/PIS | HC | - | - | - | - | - | - | - | - | - | - | - | - | HC | - | HB/HC/PIS | SB | HC/PIT/CPI | - | - | HC | - | - |
| **5ISM**  **- Chain B -**  **conformation 2** | - | - | - | - | - | HB/SB | HB/SB | - | - | - | - | - | - | - | HC/PIS/CPI | HB | - | - | - | - | - | - | - | - | - | - | HB/HC | - | - | - | HC/PIS | SB | HC/PIT | - | - | HC | - | - |
| **5KBY - Chain A** | - | - | - | - | - | SB | SB | - | - | - | - | - | - | - | HC | - | - | HC/PIS | - | - | - | - | - | - | - | HC | HB/HC | - | HC | - | HC/PIS | - | HC/PIT | - | - | HC | - | - |
| **5KBY - Chain B** | - | - | - | - | - | HB/SB | SB | - | - | - | - | - | - | - | HC | - | - | HC/PIS | - | - | - | - | - | - | - | HC | HB/HC | - | HC | - | HC/PIS | - | HC/PIT |  |  | HC |  | - |
| **5T4B - Chain A** | - | - | - | - | - | HB/SB | HB/SB | - | - | - | - | - | - | - | HC | - | - | HC/PIS | - | - | - | - | - | - | HC/PIS | - | HB/HC | - | HC | - | HB/HC | SB | HC | - | - | HC | HC | - |
| **5TKB - Chain B** | - | - | - | - | - | HB/HC/SB | HB/SB | - | - | - | - | - | - | - | HC | - | - | HC/PIS | - | - | - | - | - | - | HC/PIS | - | HB/HC | - | - | - | HB/HC | SB | HC | - | - | HC | - | - |
| **5T4E**  **- Chain A** | - | - | - | - | - | HB/SB | HB/SB | - | - | - | - | - | - | - | HC | - | - | HC/PIS | - | - | - | - | - | - | HC | HC | HB/HC | - | HC | - | HC | SB | HC | - | - | HC | - | - |
| **5T4E**  **- Chain B** | - | - | - | - | - | HB/SB | HB/SB | - | - | - | - | - | - | - | HC | - | - | HC/PIS | - | - | - | - | - | - | HC/PIS | - | HB/HC | - | HC | - | HB/HC | SB | HC | - | - | HC | - | - |
| **5T4F**  **- Chain A** | - | - | - | - | - | HB/SB | HB/SB | - | - | - | - | - | - | - | HC | - | - | HC/PIS | - | - | - | - | - | - | HC/PIS | HB | HC | - | - | - | HB/HC | SB | HC | - | - | HC | - | - |
| **5T4F**  **- Chain B** | - | - | - | - | - | HB/SB | HB/SB | - | - | - | - | - | - | - | HC | - | - | HC/PIS | - | - | - | - | - | - | HC/PIS | HC | HB/HC | - | HC | - | HB/HC | SB | HC | - | - | HC | - | - |
| **5T4H**  **- Chain A** | - | - | - | - | - | HB/HC/SB | HB/HC/SB | - | - | - | - | - | - | - | HC | - | - | HC/PIS | - | - | - | - | - | - | HC/PIS | HC | HB | - | HC | - | HB/HC | SB | HC | - | - | HC | - | - |
| **5T4H**  **-**  **Chain B** | - | - | - | - | - | HB/SB | HB/SB | - | - | - | - | - | - | - | HC | - | - | HC/PIS | - | - | - | - | - | - | HC/PIS | HC | HB | - | HC | - | HB/HC | SB | HC | - | - | HC | - | - |
| **6B1E**  **- Chain A** | - | HB | - | - | - | HB/HC/SB | HB/HC/SB | - | - | - | - | - | - | - | HC | - | - | - | - | - | - | - | - | - | - | HC | HB | - | HC | - | HC | - | HC | - | - | HC | - | - |
| **6B1E - Chain B** | - | - | - | - | - | HB/HC/SB | HB/HC/SB | - | - | - | - | - | - | - | HC | - | - | - | - | - | - | - | - | - | - | HC | HB | - | HC | - | HC | - | HC | - | - | HC | - | - |
| **6B1O - Chain A** | - | - | - | - | - | HB/SB | HB/SB | - | - | - | - | - | - | - | HC | - | - | HB | - | - | - | - | - | - | - | HC | HC | - | - | - | HB/HC | SB | HC | - | HB | HC | - | - |
| **6B1O - Chain B** | - | - | - | - | - | HB/SB | HB/SB | - | - | - | - | - | - | - | HC | - | - | HB | - | - | - | - | - | - | - | HC | HB/HC | - | HC | - | HB/HC | SB | HC | - | HB | HC | - | - |

HC: hydrophobic contact **|** PIT: π-π T-shaped **|** PIS: π-π stacking **|** HB: Hydrogen bridge **|** SB: saline bridge **|** CPI: cátion-π.
